# Emerging Monkeypox Virus Sublineage C.1 Causing Community Transmission, Vietnam, 2023

**DOI:** 10.3201/eid3011.240729

**Published:** 2024-11

**Authors:** Huynh Thi Thuy Hoa, Nguyen Thanh Dung, Le Manh Hung, Nguyen Thi Thu Hong, Vo Truong Quy, Nguyen Thi Thao, Nguyen Trong Duy, Hoang Truong, Tran Minh Hoang, Nguyen Thi Thanh, Mai Pham Hong Phuoc, Truong Ngoc Trung, Nguyen Nhut Thong, Nguyen Duc Huy, Vu Thi Kim Thoa, Vo Trong Vuong, Ngo Tan Tai, Huynh Kim Nhung, Dao Phuong Linh, Pham Thi Ngoc Thoa, Lam Minh Yen, Tran Ba Thien, Truong Hoang Chau Truc, Le Kim Thanh, Nguyen Thi Han Ny, Vo Tan Hoang, Nghiem My Ngoc, Dinh Nguyen Huy Man, Louise Thwaites, Tran Tan Thanh, Nguyen Van Vinh Chau, Guy Thwaites, Nguyen To Anh, Le Van Tan

**Affiliations:** Hospital for Tropical Diseases, Ho Chi Minh City, Vietnam (H.T.T. Hoa, N.T. Dung, L.M. Hung, V.T. Quy, N.T. Duy, H. Truong, T.M. Hoang, N.T. Thanh, M.P.H. Phuoc, T.N. Trung, N.N. Thong, N.D. Huy, V.T.K. Thoa, V.T. Vuong, N.T. Tai, H.K. Nhung, D.P. Linh, P.T.N. Thoa, N.M. Ngoc); Oxford University Clinical Research Unit, Ho Chi Minh City (N.T.T. Hong, N.T. Thao, L.M. Yen, T.B. Thien, T.H.C. Truc, L.K. Thanh, N.T.H. Ny, V.T. Hoang, L. Thwaites, T.T. Thanh, G. Thwaites, N.T. Anh, L.V. Tan); Centre for Tropical Medicine and Global Health University of Oxford, Oxford, UK (L. Thwaites, G. Thwaites, L.V. Tan); Department of Health, Ho Chi Minh City (N.V.V. Chau)

**Keywords:** viruses, mpox, monkeypox virus, sexually transmitted infections, sublinear C1, HIV co-infection, genetic changes, Vietnam

## Abstract

We studied a community cluster of 25 mpox cases in Vietnam caused by emerging monkeypox virus sublineage C.1 and imported into Vietnam through 2 independent events; 1 major cluster carried a novel APOBEC3-like mutation. Three patients died; all had advanced HIV co-infection. Viral evolution and its potential consequences should be closely monitored.

To date, most globally reported mpox sequences have come from Europe and North America, where sustained human-to-human transmission has resulted in explosive mpox outbreaks, especially in 2022 (*1*). A hallmark of the monkeypox virus (MPXV) strain responsible for the ongoing global outbreaks is its high evolution rate, which is driven by the host APOBEC3 (apolipoprotein B mRNA editing enzyme, catalytic polypeptide 3) deaminases, causing a dinucleotide change from TC to TT (*2*). In addition, persons with advanced HIV might experience more severe outcomes (*3*) and delayed viral clearance, resulting in the emergence of new variants, as has been observed with SARS-CoV-2 (*4*). However, this possibility has not been well studied for MPXV infection (*5*).

Vietnam reported its first mpox cases in late 2022 in 2 female travelers returning from United Arab Emirates (*6*). No additional cases were reported until September 2023, when mpox was diagnosed in a 33-year-old man in Dong Nai Province in southern Vietnam (*7*). This case marked the start of ongoing community transmission in Vietnam, where the mpox vaccine has not been deployed.

Despite the ongoing challenges of mpox, existing literature has been dominated by reports from Europe and North America, where most cases have been reported (*1*). We therefore studied the longitudinal clinical, laboratory, and virological features in mpox patients admitted to a tertiary referral hospital in Ho Chi Minh City, Vietnam, in 2023. We also sought to study virus evolution in persons with advanced HIV over the course of hospitalization.

## The Study

In Vietnam, all persons with mpox are subject to isolation at a designated healthcare center for >14 days or until clinical manifestations resolve. This study was conducted at the Hospital for Tropical Diseases (HTD) in Ho Chi Minh City. HTD is a tertiary referral infectious diseases hospital and a designated hospital for isolating and treating mpox patients in Ho Chi Minh City, which has a population of ≈10 million persons. The study was approved by the HTD Institutional Review Board and Oxford Tropical Research Ethics Committee. Written informed consent was obtained from study participants. 

We collected laboratory and clinical data from recruited patients at admission, as well as lesion swab samples for analysis. Where relevant, cerebrospinal fluid (CSF) samples, endotracheal aspirate (ETA) samples, and follow-up lesion swab samples were also collected from patients with clinical complications. Daily clinical follow-up of patients took place during hospitalization, including assessment for new and evolving lesions.

We diagnosed MPXV using LightMix Modular Mpox PCR (TIB Molbiol, https://www.tib-molbiol.de). We generated MPXV genomes directly from representatives of admission lesion swabs and other sample types (if available) with a PCR cycle threshold (Ct) value <30 using a metagenomics-based approach (*6*) (Appendix).

During September–December 2023, a total of 54 mpox patients were admitted to HTD, and 25 (46%) participated in the clinical studies. The participants were 22–49 (median 31) years of age; 88% (22/25) were men who have sex with men (Table). No patients reported recent travel history outside of Vietnam. HIV infection was documented in 21 (84%) persons; median CD4 cell count was 14 (range 1–579) cells/µL. Of those 21 patients, 16 (76%) were receiving antiretroviral therapy. Concurrent sexually transmitted diseases were documented in 12/24 (50%) persons; the most common were syphilis (n = 9) and gonorrhea (n = 5) (Table).

**Table:** Baseline characteristics of 25 participants in study of emerging monkeypox virus sublineage C.1 causing community transmission, Vietnam, 2023*

Characteristic	Value
Median age, y (range)	31 (22–49)
From Ho Chi Minh City	20/25 (80)
Median time from symptom onset to admission, d (range)	6 (4–30)
Median hospitalization, d (range)	14 (11–43)
Sexual activity within 21 d before illness	18/25 (72)
Travel abroad within 21 d before illness	0/25 (0)
HIV-positive	21/25 (84)
Receiving antiretroviral therapy	16/21 (76)
Gonorrhea	5/25 (20)
Syphilis	9/25 (36)
Symptoms	
Fever	14/25 (56)
Sore throat	5/25 (20)
Myalgia	2/25 (8)
Headache	1/25 (4)
Fatigue	2/25 (8)
Oral pain	2/25 (8)
Diarrhea	2/25 (8)
Rectal pain	6/25 (24)
Pain with swallowing	2/25 (8)
Difficulty swallowing	1/25 (4)
Lymphadenopathy	16/25 (64)
Cervical	9/16 (56)
Inguinal	9/16 (56)
Median temperature, °C (range)	37 (37–38.5)
Median heart rate, bpm (range)	85 (75−130)
Median respiratory rate, bpm (range)	20 (18–22)
Median arterial pressure, mm Hg (range)	83 (73–107)
Laboratory findings, median (range)/(reference range)	
CD4+ count, cells/µL	451 (1–956)/(500–1600)
leukocyte count, 10^9^ cells/L	9.98 (5.53–17.08)/(4.5–11)
Hemoglobin, g/L	147 (11–174)/(130–160)
Platelet count, 10^9^/L	256 (131–473)/(140–440)
Glucose, mg/dL	83 (6–138)/(70–100)
Aspartate aminotransferase, U/L	24 (15–48)/(0–40)
Alanine aminotransferase, U/L	35 (11–85)/(0–40)
Creatinine, µmol/L	89 (70–147)/(53–120)
No. lesions	
1–5	1/25 (4)
6–25	11/25 (44)
26–100	12/25 (48)
>250	1/25 (4)
Lesion position	
Arm	20/25 (80)
Leg	19/25 (76)
Genitals	19/25 (76)
Oral mucosa	5/25 (20)
Perianal area	15/25 (60)
Lesion characteristics	
Macule	5/25 (20)
Papule	6/25 (24)
Early vesicle	11/25 (44)
Small pustule	21/25 (84)
Umbilicated pustule	12/25 (48)
Ulcerated lesion	6/25 (24)
Crusting of a mature lesion	10/25 (40)
Partially removed scab	3/25 (12)

*Values are no. (%) unless otherwise indicated. All patients were assigned male sex at birth; 24/25 reported having sex with men (2 men identified as bisexual). No participants had been vaccinated for mpox or had a tecovitimat prescription.

**Figure 1:**
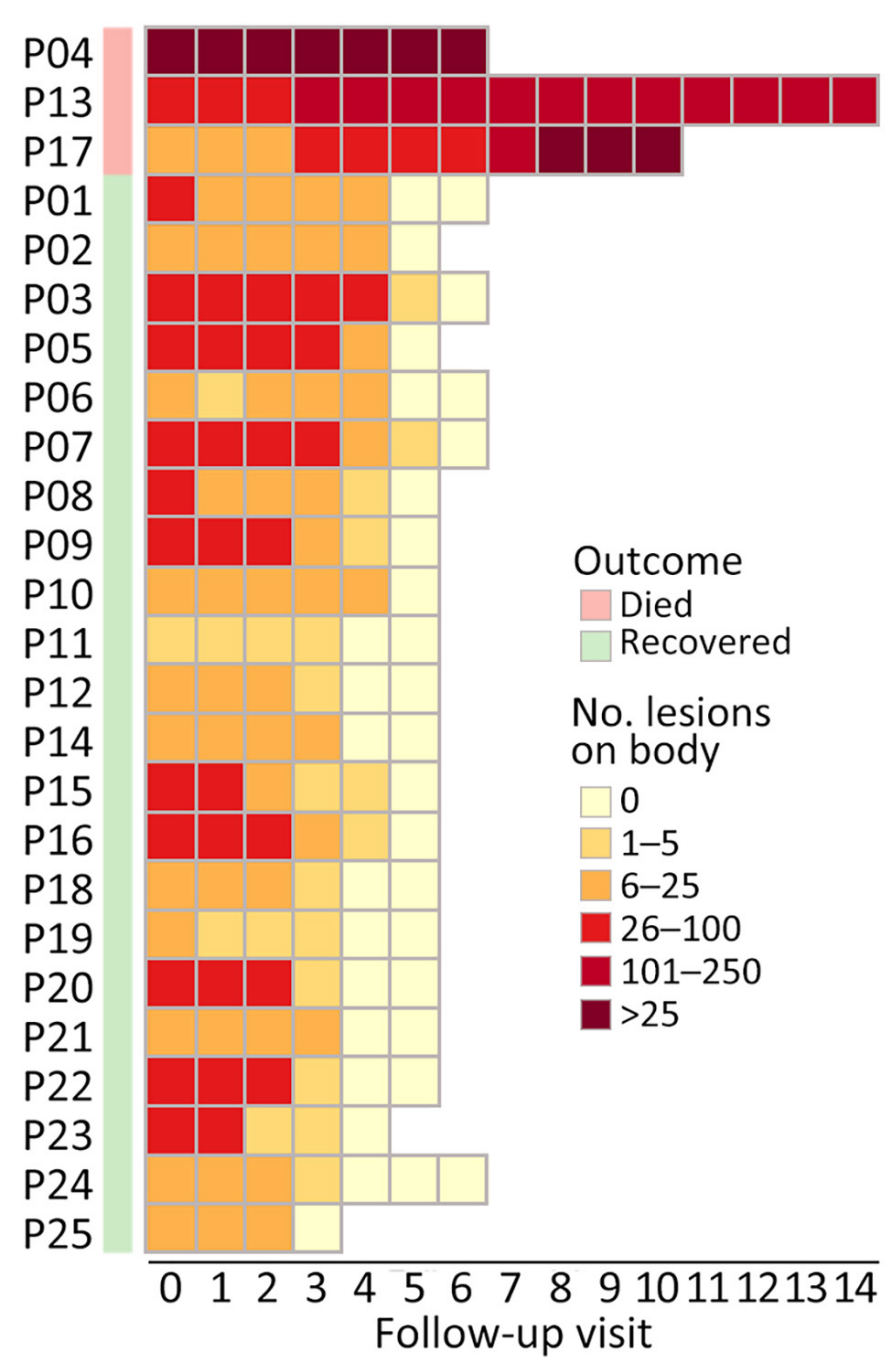
Heat map showing changes in the number of skin lesions over the course of hospitalization of 3 fatal (P04, P13, and P17) and 22 nonfatal cases in study of emerging monkeypox virus sublineage C.1 causing community transmission, Vietnam, 2023. Median follow-up was 3 (range 3–5) days. P, patient number.

**Figure 2:**
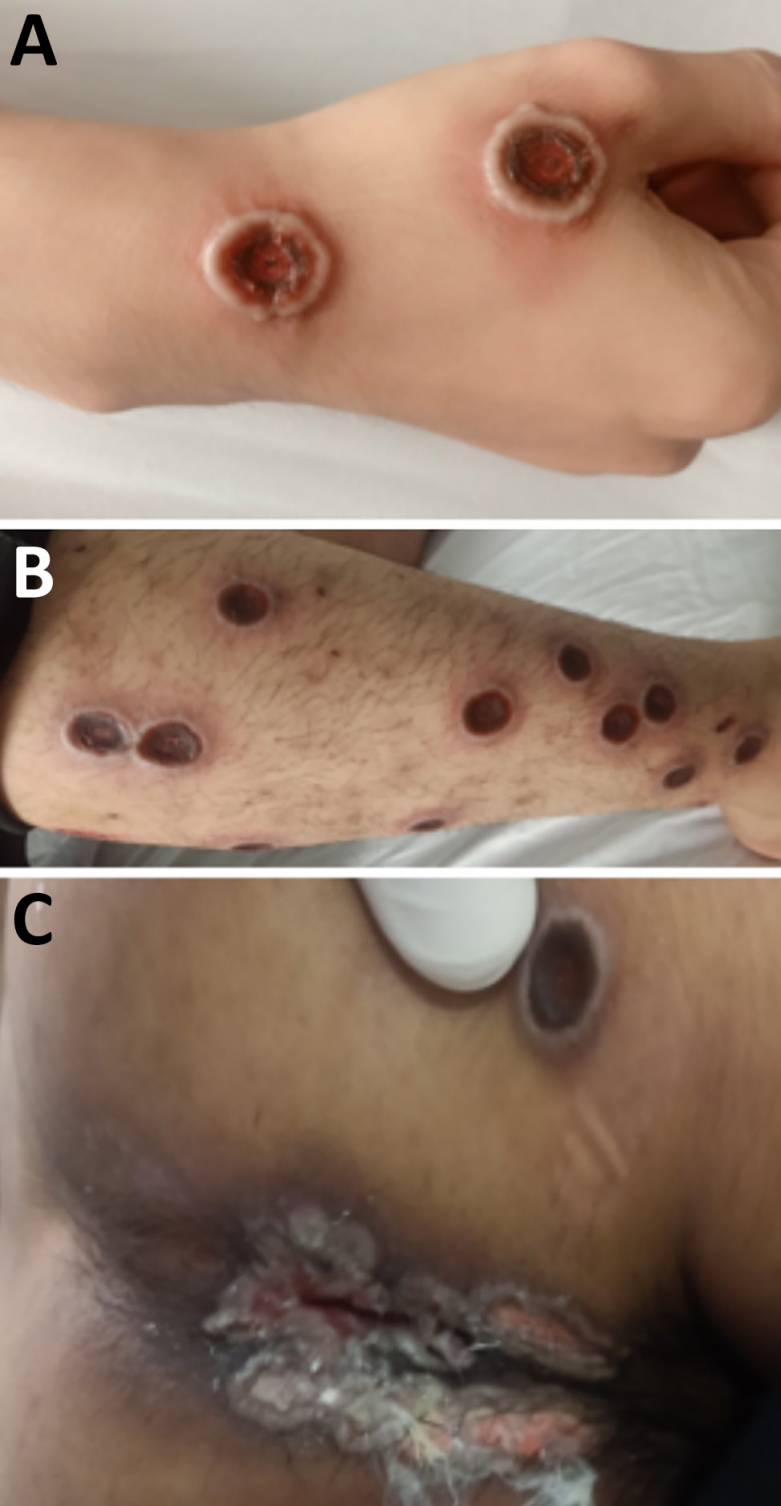
Natural progression of skin lesions of mpox patient who died in study of emerging monkeypox virus sublineage C.1 causing community transmission, Vietnam, 2023. Skin lesions shown are on the right hand (A), right leg (B), and perianal and buttock areas (C) of a patient with a CD4 cell count of 16 cells/µL.

A total of 3 patients had severe disease that required intensive care unit admission for septic shock; the patients subsequently died (Appendix Table 1). Those patients were all young adults with advanced HIV infection (low CD4 cell counts) (Appendix Table 1); only 1 was receiving antiretroviral therapy. One patient had other opportunistic infections (tuberculosis and pneumocystis pneumonia) (Appendix Table 1), and MPXV DNA was detected in both ETA (PCR Ct 17.67) and CSF (PCR Ct 33.82). Lumbar puncture was indicated in this patient because he began hallucinating. We did not perform testing for other pathogens in the CSF, but the CSF parameters were with reference ranges (data not shown). All patients who died had prominent skin lesions (confluence and necrosis) (Figures 1, 2) that remained unhealed. The remaining 22 (88%) patients made a full recovery; all lesions resolved before hospital discharge.

**Figure 3:**
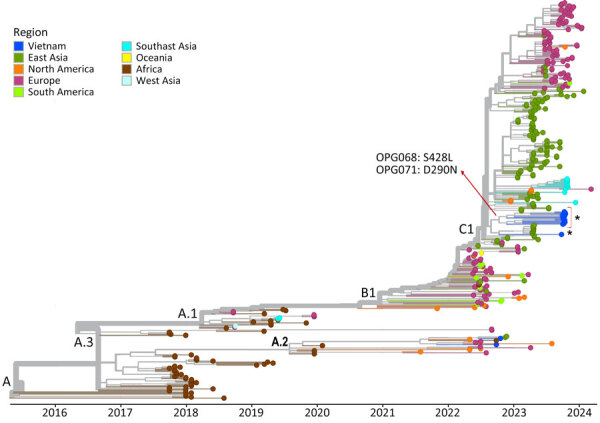
Assessment of genetic diversity of the monkeypox virus genomes obtained in study of emerging monkeypox virus sublineage C.1 causing community transmission, Vietnam, 2023. Time-scaled phylogenetic tree of 14 Vietnam monkeypox virus genomes from this study (asterisk) highlights 2 nonsynonymous substitutions shared by 13 sequences (red arrow), with substitution OPG068: S428L uniquely found in those 13 sequences. D, aspartate; L, leucine; N, asparagine; OPG, orthopoxvirus gene; S, serine.

We successfully obtained 14 complete-genome sequences from 14 patients (Appendix Table 1). Phylogenetically, they all exhibited a close relatedness with sublineage C.1 viruses of MPXV clade IIb but formed 2 separate clusters, including 1 cluster of 13 sequences sharing 2 nonsynonymous substitutions (Figure 3). Pairwise analysis identified 12 nonsynonymous mutations (2 APOBEC3-like and 10 non–APOBEC3-like) in the 14 Vietnam sequences but not in the global MPXV dataset as of July 29, 2024 (Appendix Figure 1). The 2 APOBEC3-like mutations were detected in 2/14 (14.3%) sequences and 13/14 (92.9%) sequences, as compared with 1/14 (7.1%) sequence for the 10 non–APOBEC3-like mutations (Appendix Figure 1). Subsequently, Sanger sequencing confirmed the presence of the APOBEC3-like mutation (OPG068: S428L) coexisting in 13/14 sequences in the original samples (Appendix Figure 2). We did not perform Sanger sequencing for the remaining 11 sporadic mutations because materials were unavailable. The 12 substitutions were mostly located in functional proteins concerning virus assembly, virulence, or replication (Appendix Table 3).

Of the patients who died, 1 person had 4 longitudinal lesion swab samples and an ETA available for intrahost evolution analysis. We found evidence of nonsynonymous substitutions (n = 2) and single-nucleotide polymorphisms (n = 6) in the metagenomics datasets (Appendix Table 4). However, subsequent Sanger sequencing failed to confirm their presence in the original samples (Appendix Figure 3).

Our findings emphasize that, although MPXV infections are usually self-limiting, severe clinical complications and death can occur, especially in persons with advanced HIV (*3*,*8*). Detecting MPXV in ETA and CSF samples is unusual, although it has been reported previously (*3*), and this finding supports further study of mpox pathogenesis.

The responsible viruses belonged to sublineage C.1, lineage B.1 of clade IIb, and were imported into Vietnam through 2 independent events, as demonstrated by their phylogenetically forming into 2 different clusters. Sublineage C.1 has only recently emerged and caused local transmission in China (*9*). In addition, C.1 sequences from various countries in Asia, Europe, and the Americas have been deposited to GISAID (https://www.gisaid.org), demonstrating its global dispersal. Those collective findings point to a rapid evolution of MPXV, of which the host APOBEC3 has been shown to be a main driver (*2*). Alternatively, immune suppression or antivirals might also enable intrahost evolution, as observed in a recent study (*5*). Similar findings were documented in our metagenomics datasets of longitudinal samples. However, subsequent Sanger sequencing failed to confirm those original findings, likely attributed to sequencing artifacts, emphasizing the importance subsequent Sanger sequencing–based confirmatory experiments.

The tight cluster on the global phylogenetic tree of the 13 sequences sharing 2 nonsynonymous substitutions suggested that those patients shared a transmission network, supporting findings from a recent report (*10*). Because direct skin-to-skin contact plays a key role in MPXV transmission, public education campaigns should raise awareness about behaviors that increase the risk for MPXV exposure (*11*). Vaccination remains the most effective tool to control mpox outbreaks (*12*).

## Conclusions

We report the clinical, laboratory, and virological findings in 25 mpox patients infected with an emerging sublineage C.1 that was imported into Vietnam through 2 independent events; 1 major cluster carried a novel APOBEC3-like mutation concerning virus assembly. MPXV evolution and its potential consequences should be closely monitored. Clinicians should be aware of unusual skin lesions in patients with advanced HIV.

Appendix Additional information about emerging monkeypox virus sublineage C.1 causing community transmission, Vietnam, 2023
