# Supplementary material for: Emerging Monkeypox Virus Sublineage C.1 Causing Community Transmission, Vietnam, 2023
Source: Emerg Infect Dis. 2024 Nov;30(11):2385–90. doi: 10.3201/eid3011.240729 (PMC11521191; doi:10.3201/eid3011.240729)
Supplement: Appendix — Additional information about emerging monkeypox virus sublineage C.1 causing community transmission, Vietnam, 2023 [file 24-0729-Techapp-s1.pdf]

Article DOI: https://doi.org/10.3201/eid3011.240729

EID cannot ensure accessibility for Supplemental Materials supplied by authors. Readers who have difficulty accessing supplementary content should contact the authors for assistance.

Emerging Monkeypox Virus Sublineage C.1 Causing Community Transmission, Vietnam, 2023

Appendix

Genome amplification and sequencing

MPXV whole-genome sequencing was carried out using our established metagenomics based approach that has delivered consistent results for emerging viruses, including MPXV (*1*–*4*). In brief, 100 µL of sample were pre-treated with 20 U of turbo DNase and 2U RNase I (Ambion-Life Technology, ThermoFisher) at 37°C for 30 min to reduce host genetic background. Nucleic acids (NA) were then isolated from the pre-treated samples using DNeasy Blood & Tissue Kit (QIAGEN), and eluted in 50 µL of the elution buffer provided with the extraction kit. Next, the isolated NA was subjected to a pre-amplification step to enrich for viral NA using a non-ribosomal random PCR assay with primers designed to preferentially bind to viral NA but not human ribosomal RNA (*1*,*5*). The random PCR procedure was carried out as previously described (*1*,*2*). In brief, double-stranded DNA was synthesized from the isolated nucleic acids using a set of 96 non-ribosomal primer (Appendix Table 5), followed by a random amplification step using FR20RV primer (Appendix Table 5). The amplified products were then cleaned up and used as input for whole-genome sequencing. For this step, Illumina COVIDSeq reagents (Illumina) were used to generate DNA library, following manufacturer's instructions. The concentration and size of the constructed library were accessed using Qubit dsDNA HS kit (Invitrogen) and Agilent High Sensitivity D1000 kit (Aligent Technologies), respectively. Finally, the prepared library was sequenced using MiSeq Reagent kit V3 (150 cycles) or Miseq Nano kit V2 (300 cycles) (Illumina) in a Miseq platform.

Sequence assembly

A reference-based mapping strategy available in Geneiuos Prime 2022.2.2 (https://www.geneious.com) was applied to generate the consensuses from the obtained sequencing reads with consensus calling criteria of ≥5 reads (*6*). For this analysis, we use a MPXV sequence (LC760460.1) as a reference. The obtained consensuses were further manually edited to clarify the ambiguous positions (if any), and to exclude bases with low quality (i.e., a Phred quality score of <20).

Assessment of intra-host evolution over the course of illness and PCR confirmatory testing

For investigation of within host evolution over the course of illness, individual positions across the genome were carefully inspected to identify the potential changes leading to non-synonymous substitutions. For this analysis, areas with low sequencing depth (i.e., <5 reads with high quality reflected by a Phred quality score of > = 30) were also taken into account during the initial screening step. To rule out potential biases introduced by metagenomisc associated steps, potential non-synonymous substitutions were then subjected to confirmatory testing using PCR followed by Sanger sequencing using with primers designed to capture the sequence of interest (Appendix Table 6). Only mutations confirmed by both methods were considered as real substitutions. Synonymous substitutions were not investigated in this study.

Phylogenetic and mutation analysis

Here we focused our analysis on sublineage C.1. Accordingly, we retrieved all non-redundant whole-genome sequences of this sublineage deposited to GISAID (https://www.epicov.org/epi3/frontend#3b750a) or NCBI Virus (taxid: 10244) as of March 28th 2024. This resulted in a total of 223 whole-genome sequences of sublineage C.1. For non-C.1 lineages, we used 148 whole-genome sequences representing for geographic regions from which those viruses were isolated as indicated by Nexstrain, including (lineage A (n = 43), sublineage A.1 (n = 20), sublineage A.2 (n = 18), lineage sub A.3 (n = 7), sublineage B.1 (n = 60). Consequently, a total of 385 whole-genome sequence (223 C.1, 148 non-C.1. and 14 from the present study, Appendix Table 7) were included for phylogenetic analysis. A time resolved

phylogenetic tree was then reconstructed using Nextstrain pipeline with Augur v24.2.3. A lineage-A sequence (Accession number: NC_063383.1) was used to root the tree.

For detection of novel substitutions, we used sequence alignment of sublineage C.1 viruses. We performed the analysis using Nextclade v3.3.1 (https://clades.nextstrain.org/), and a lineage-A sequence (Accession number: NC_063383.1) was used as a reference sequence. Additionally, a global MPXV dataset consisting of 10544 non-redundant sequences retrieved GISAID and the NCBI GenBank (29 July 2024) was assembled to further exclude the presence of such potential novel substitutions in non-C.1 sublineage sequences.

References

1. Nguyen AT, Tran TT, Hoang VM, Nghiem NM, Le NN, Le TT, et al. Development and evaluation of a non-ribosomal random PCR and next-generation sequencing based assay for detection and sequencing of hand, foot and mouth disease pathogens. Virol J. 2016;13:125. PubMed https://doi.org/10.1186/s12985-016-0580-9

2. Anh NT, Hong NTT, Nhu LNT, Thanh TT, Lau CY, Limmathurotsakul D, et al. Viruses in Vietnamese patients presenting with community-acquired sepsis of unknown cause. J Clin Microbiol. 2019;57:e00386-19. PubMed https://doi.org/10.1128/JCM.00386-19

3. Dung NT, Hung LM, Hoa HTT, Nga LH, Hong NTT, Thuong TC, et al. Monkeypox virus infection in 2 female travelers returning to Vietnam from Dubai, United Arab Emirates, 2022. Emerg Infect Dis. 2023;29:778–81. PubMed https://doi.org/10.3201/eid2904.221835

4. Anh NT, Nhu LNT, Van HMT, Hong NTT, Thanh TT, Hang VTT, et al. Emerging coxsackievirus A6 causing hand, foot and mouth disease, Vietnam. Emerg Infect Dis. 2018;24:654–62. PubMed https://doi.org/10.3201/eid2404.171298

5. Endoh D, Mizutani T, Kirisawa R, Maki Y, Saito H, Kon Y, et al. Species-independent detection of RNA virus by representational difference analysis using non-ribosomal hexanucleotides for reverse transcription. Nucleic Acids Res. 2005;33:e65. PubMed https://doi.org/10.1093/nar/gni064

6. Ladner JT, Beitzel B, Chain PS, Davenport MG, Donaldson EF, Frieman M, et al.; Threat Characterization Consortium. Standards for sequencing viral genomes in the era of high-throughput sequencing. MBio. 2014;5:e01360–14. PubMed https://doi.org/10.1128/mBio.01360-14

Appendix Table 1. Detailed clinical and laboratory findings of the 3 fatal cases in study of emerging monkeypox virus sublineage C.1 causing community transmission, Vietnam, 2023*

Characteristic	Patient 04	Patient 13	Patient 17
Age, y	29	27	27
Sex at birth	M	M	M
Duration of hospital stay, d	19	13	29
HIV infection	Yes	Yes	Yes
CD4 cell count, cells/μL	01	16	05
Antiretroviral therapy	No	No	Yes
Mechanical ventilation	Yes	Yes	Yes
Septic shock	Yes	Yes	Yes
Anemia	Yes	Yes	No
Acute respiratory distress syndrome	Yes	Yes	Yes
Acute renal injury	Yes	No	Yes
Acute liver dysfunction	Yes	Yes	Yes
Ocular infection	Yes	Yes	Unknown
No. skin lesions at baseline	>250	101–250	>250
Lesion characteristics			
Necrotizing	Yes	Yes	Yes
Confluent	Yes	Yes	Yes
Size, mm	5–7	20–25	3–7
Comorbidity†	Yes	No	Yes
Tuberculosis	Positive‡	Negative	Not done
Syphilis§	Negative	Postive	Not done
Pneumocystis jirovecii¶	Positive	negative	Not done
Others	*Candida albicans*	No	*Candida spp*

*Patient codes are consistent with those shown in Appendix Figure 1. Virus from P17 was not sequenced.
†Results of routine culture.
‡Gastrointestinal and military tuberculosis by Ziehl–Neelsen smear microscopy examination of stool and sputum samples.
§By rapid test.
¶By PCR diagnosis.

Appendix Table 2. Summary of sequencing results of 14 genome sequences from 14 patients

Sample ID	Sample type	Number of raw reads	Number of mapped reads	Mean sequencing depth (min-max)	% Genome coverage
P01	Lesion swab	2,947,694	1,631,089	1339 (5–17987)	96.07
P02	Lesion swab	3,375,932	2,528,234	963.4 (5–30081)	95.49
P03	Lesion swab	2,018,174	726,272	274.5 (5–9138)	90.29
P04	Lesion swab	3,256,816	2,684,010	1050.7 (5–38317)	96.23
P05	Lesion swab	3,086,716	2,016,767	769.5 (5–26302)	95.27
P06	Lesion swab	3,064,754	309,579	116.8 (5–6485)	72.93
P07	Lesion swab	2,978,534	352,866	132.8 (5–5172)	80.80
P08	Lesion swab	2,778,924	401,856	151 (5–5642)	79.28
P10	Lesion swab	3,787,078	3,453,213	1330 (5–56824)	95.83
P11	Lesion swab	2,199,790	1,338,296	508.1 (5–13463)	89.86
P09	Lesion swab	2,773,716	2,365,381	915.4 (5–43935)	96.46
P14	Lesion swab	877,648	546,075	205.8 (5–7540)	84.46
P13	Lesion swab	3,848,392	3,462,380	1343.1 (5–48465)	98.44
P15	Lesion swab	3,737,842	2,738,850	1053.3 (5–126305)	97.32

Appendix Table 3. Frequency, location and associated protein functions of the 12 novel mutations

Novel mutation	Protein Function	APOBEC3-like mutation	Frequency
OPG027:E104K	Host Modulation	Yes	2/14
OPG047:D462Y	Unknown	No	1/14
OPG063:T225S	Transcription	No	1/14
OPG063:N239S	Transcription	No	1/14
OPG068:S428L	Virus Assembly/Budding	Yes	13/14
OPG087:P186T	Transcriptionssss	No	1/14
OPG102:T110A	Transcription	No	1/14
OPG108:N117K	Virulence	No	1/14
OPG118:N76Y	Transcription	No	1/14
OPG138:S170P	Morphogenesis	No	1/14
OPG160:S236P	Morphogenesis	No	1/14
OPG189:I514S	Virulence	No	1/14

Appendix Table 4. Results of intra-host evolution investigation of metagenomics reads in samples collected over the course of illness of an advanced HIV patient

Category	Type	Lesion swab (10/2/2023)		Lesion swab(10/9/2023)		Lesion swab (10/16/2023)		BAL (10/19/2023)		Lesion swab (10/11/2023)	
OPG189:I514S	Substitution	Ile to Ser		Ile to Ser		Ile to Ser		Ile to Ser		Ile to Ser	
	Ratio (%)	51	49	22	78	24	76	26	74	22	78
	Sequencing depth	183	176	14	49	29	92	34	96	29	103
OPG187:K233M	Substitution	NA	NA	NA	NA	NA	NA	NA	NA	Lys to Met	
	Ratio (%)	NA	NA	NA	NA	NA	NA	NA	NA	46	54
	Sequencing depth	NA	NA	NA	NA	NA	NA	NA	NA	50	59
OPG060:M147I	Substitution	NA	NA	NA	NA	NA	NA	Ile to Met		NA	NA
	Ratio (%)	NA	NA	NA	NA	NA	NA	0	100	NA	NA
	Sequencing depth	NA	NA	NA	NA	NA	NA	0	4	NA	NA
OPG164:S63C	Substitution	NA	NA	NA	NA	NA	NA	Ser to Cys		NA	NA
	Ratio (%)	NA	NA	NA	NA	NA	NA	0	100	NA	NA
	Sequencing depth	NA	NA	NA	NA	NA	NA	0	2	NA	NA

*Two nonsynonymous substitutions detected in the BAL collected on 19 October 2023 are in red. Positions carrying single nucleotide polymorphism are indicated by blue. The results of Sanger sequencing of those corresponding positions shown in Appendix Figure 3. NA: indicate positions where no changes were recorded. None of the lesion swabs were collected from the same rash or area.

Appendix Table 5. Primer sequences used for non-ribosomal random PCR

Primer	Sequence of primer (5′-3′)	Primer	Sequence of primer (5′-3′)	Primer	Sequence of primer(5′-3′)
FR20RV	GCCGGAGCTCTGCAGATATC	33	GCCGGAGCTCTGCAGATATCTAGTCG	66	GCCGGAGCTCTGCAGATATCTAACGC
1	GCCGGAGCTCTGCAGATATCGATATC	34	GCCGGAGCTCTGCAGATATCGTAGAC	67	GCCGGAGCTCTGCAGATATCGGTCAT
2	GCCGGAGCTCTGCAGATATCTAGTAT	35	GCCGGAGCTCTGCAGATATCCTATAG	68	GCCGGAGCTCTGCAGATATCCTCATA
3	GCCGGAGCTCTGCAGATATCTATAGT	36	GCCGGAGCTCTGCAGATATCTAGCTA	69	GCCGGAGCTCTGCAGATATCAATTTG
4	GCCGGAGCTCTGCAGATATCTATATA	37	GCCGGAGCTCTGCAGATATCACTACT	70	GCCGGAGCTCTGCAGATATCCTGGTA
5	GCCGGAGCTCTGCAGATATCATACTA	38	GCCGGAGCTCTGCAGATATCTAACGA	71	GCCGGAGCTCTGCAGATATCTTCATG
6	GCCGGAGCTCTGCAGATATCATATAT	39	GCCGGAGCTCTGCAGATATCCGACTA	72	GCCGGAGCTCTGCAGATATCGCGATA
7	GCCGGAGCTCTGCAGATATCGTGCAC	40	GCCGGAGCTCTGCAGATATCTACTAG	73	GCCGGAGCTCTGCAGATATCACTAAG
8	GCCGGAGCTCTGCAGATATCACTATA	41	GCCGGAGCTCTGCAGATATCAGTAGT	74	GCCGGAGCTCTGCAGATATCGCATAC
9	GCCGGAGCTCTGCAGATATCCGTAAT	42	GCCGGAGCTCTGCAGATATCGTTAAC	75	GCCGGAGCTCTGCAGATATCCAATAT
10	GCCGGAGCTCTGCAGATATCCTATAC	43	GCCGGAGCTCTGCAGATATCGTCTAC	76	GCCGGAGCTCTGCAGATATCACCGTA
11	GCCGGAGCTCTGCAGATATCTATACG	44	GCCGGAGCTCTGCAGATATCTACAAG	77	GCCGGAGCTCTGCAGATATCGTGCTA
12	GCCGGAGCTCTGCAGATATCTATGCG	45	GCCGGAGCTCTGCAGATATCTACCAG	78	GCCGGAGCTCTGCAGATATCACGCTA
13	GCCGGAGCTCTGCAGATATCGATACT	46	GCCGGAGCTCTGCAGATATCTGGATT	79	GCCGGAGCTCTGCAGATATCATGTCG
14	GCCGGAGCTCTGCAGATATCCGTATA	47	GCCGGAGCTCTGCAGATATCTCGTTA	80	GCCGGAGCTCTGCAGATATCAGCTTA
15	GCCGGAGCTCTGCAGATATCGTATAG	48	GCCGGAGCTCTGCAGATATCATAGTA	81	GCCGGAGCTCTGCAGATATCCGACAT
16	GCCGGAGCTCTGCAGATATCCGGTTA	49	GCCGGAGCTCTGCAGATATCATAGTC	82	GCCGGAGCTCTGCAGATATCGCTATA
17	GCCGGAGCTCTGCAGATATCAATAGT	50	GCCGGAGCTCTGCAGATATCCTAGTA	83	GCCGGAGCTCTGCAGATATCGCTATG
18	GCCGGAGCTCTGCAGATATCCGCATA	51	GCCGGAGCTCTGCAGATATCGTACTA	84	GCCGGAGCTCTGCAGATATCTGTAAG
19	GCCGGAGCTCTGCAGATATCATTACG	52	GCCGGAGCTCTGCAGATATCTAAGTT	85	GCCGGAGCTCTGCAGATATCAACTTA
20	GCCGGAGCTCTGCAGATATCTTAACA	53	GCCGGAGCTCTGCAGATATCATATCC	86	GCCGGAGCTCTGCAGATATCATAACG
21	GCCGGAGCTCTGCAGATATCAGTATC	54	GCCGGAGCTCTGCAGATATCTCGATA	87	GCCGGAGCTCTGCAGATATCATGTTA
22	GCCGGAGCTCTGCAGATATCTGTTAA	55	GCCGGAGCTCTGCAGATATCGTACCA	88	GCCGGAGCTCTGCAGATATCTGGTAT
23	GCCGGAGCTCTGCAGATATCACTATT	56	GCCGGAGCTCTGCAGATATCGTATCA	89	GCCGGAGCTCTGCAGATATCTGCGTA
24	GCCGGAGCTCTGCAGATATCTAACCG	57	GCCGGAGCTCTGCAGATATCATACTC	90	GCCGGAGCTCTGCAGATATCGGATAT
25	GCCGGAGCTCTGCAGATATCCGATAT	58	GCCGGAGCTCTGCAGATATCACATTA	91	GCCGGAGCTCTGCAGATATCCATAGC
26	GCCGGAGCTCTGCAGATATCGTATAC	59	GCCGGAGCTCTGCAGATATCATATTG	92	GCCGGAGCTCTGCAGATATCCATACT
27	GCCGGAGCTCTGCAGATATCAATCCA	60	GCCGGAGCTCTGCAGATATCCGTCTA	93	GCCGGAGCTCTGCAGATATCCGGATA
28	GCCGGAGCTCTGCAGATATCTAGCAC	61	GCCGGAGCTCTGCAGATATCCTTAGT	94	GCCGGAGCTCTGCAGATATCTTACTA
29	GCCGGAGCTCTGCAGATATCATATCG	62	GCCGGAGCTCTGCAGATATCCTTACA	95	GCCGGAGCTCTGCAGATATCACTCGT
30	GCCGGAGCTCTGCAGATATCAATATT	63	GCCGGAGCTCTGCAGATATCTTATGC	96	GCCGGAGCTCTGCAGATATCTAAGGT
31	GCCGGAGCTCTGCAGATATCTATAGC	64	GCCGGAGCTCTGCAGATATCATACGC		
32	GCCGGAGCTCTGCAGATATCCTTGTA	65	GCCGGAGCTCTGCAGATATCCGCTTA		

Appendix Table 6. Primer sequences used for confirmatory experiments

Mutations	Primer direction	Oligo sequences (5′-3′)
OPG060:M147I	Forward	GTTAGACAACACACCGACGA
	Reverse	TCAATATCTCCTTCCTGGGT
OPG068:S428L	Forward	ACGAGTGCTGCGGACATTAA
	Reverse	ACGAGCCAATTGTCCGTCTA
OPG164:S63C	Forward	ACCGTAGTTGCGGGAACAA
	Reverse	ACCAGCGAAGCTATCATCGT
OPG187:K233M	Forward	TCTAGACGTGGTGATCTAGA
	Reverse	TACTCGACGAAACTCGTCGT
OPG189:I514S	Forward	TGAGATACGGTAGACATCCT
	Reverse	ACGACCATTTATCTCCAGCA

Appendix Table 7. List of sequences used for phylogenetic analysis

Accession number	Source	Date of collection	Country	Host	Clade	Lineage	Analysis
NC_063383.1	GenBank	2018–08–01	Nigeria	Human	IIb	A	phylogeny
OR500078.1	GenBank	2022–06–29	Romania	Human	IIb	B.1.1	phylogeny
OR500080.1	GenBank	2022–08–16	Romania	Human	IIb	B.1.1	phylogeny
OR500084.1	GenBank	2023–01–05	Romania	Human	IIb	B.1.1	phylogeny
OR500085.1	GenBank	2023–01–26	Romania	Human	IIb	B.1.1	phylogeny
OR464192.1	GenBank	2022–12–01	USA	Human	IIb	B.1	phylogeny
OR459776.1	GenBank	2022–11–15	South Korea	Human	IIb	A.2.1	phylogeny
OR459777.1	GenBank	2022–11–22	South Korea	Human	IIb	A.2.1	phylogeny
OR455100.1	GenBank	2023–08–01	USA	Human	IIb	A.2.1	phylogeny
OR427338.1	GenBank	2023–01–31	USA	Human	IIb	B.1.2	phylogeny
OR427343.1	GenBank	2023–03–01	USA	Human	IIb	B.1.2	phylogeny
OR264418.1	GenBank	2022–07–05	Australia	Human	IIb	B.1	phylogeny
OR264422.1	GenBank	2022–07–13	Australia	Human	IIb	B.1	phylogeny
OR126132.1	GenBank	2023–03–23	Spain	Human	IIb	B.1.4	phylogeny
OR113690.1	GenBank	2023–04–05	Austria	Human	IIb	A.2.2	phylogeny
OR086938.1	GenBank	2022–06–29	Belgium	Human	IIb	B.1	phylogeny
OQ957072.1	GenBank	2023–02–16	Colombia	Human	IIb	B.1.11	phylogeny
OQ773459.1	GenBank	2022–07–01	Germany	Human	IIb	B.1	phylogeny
OQ773518.1	GenBank	2022–07–31	Germany	Human	IIb	B.1	phylogeny
OQ721951.1	GenBank	2023–03–01	Taiwan	Human	IIb	B.1	phylogeny
OQ721953.1	GenBank	2023–03–01	Taiwan	Human	IIb	B.1.3	phylogeny
OQ672572.1	GenBank	2023–01–26	Belgium	Human	IIb	B.1	phylogeny
OQ672573.1	GenBank	2023–01–26	Belgium	Human	IIb	B.1	phylogeny
OQ468900.1	GenBank	2022–07–21	USA	Human	IIb	B.1	phylogeny
OQ451903.1	GenBank	2023–01–23	Germany	Human	IIb	B.1.3	phylogeny
OQ451904.1	GenBank	2023–01–23	Germany	Human	IIb	B.1.3	phylogeny
OQ411312.1	GenBank	2022–06–08	Switzerland	Human	IIb	B.1	phylogeny
OQ261710.1	GenBank	2022–10–21	Colombia	Human	IIb	B.1.10	phylogeny
OP535312.1	GenBank	2018–02–01	Nigeria	Human	IIb	A	phylogeny
OP535313.1	GenBank	2018–01–31	Nigeria	Human	IIb	A	phylogeny
OP535314.1	GenBank	2018–01–31	Nigeria	Human	IIb	A	phylogeny
OP535315.1	GenBank	2018–02–28	Nigeria	Human	IIb	A	phylogeny
OP535316.1	GenBank	2018–02–01	Nigeria	Human	IIb	A	phylogeny
OP535317.1	GenBank	2018–01–31	Nigeria	Human	IIb	A	phylogeny
OP535318.1	GenBank	2017–12–31	Nigeria	Human	IIb	A	phylogeny
OP535319.1	GenBank	2017–12–31	Nigeria	Human	IIb	A	phylogeny
OP535320.1	GenBank	2017–11–01	Nigeria	Human	IIb	A	phylogeny
OP535321.1	GenBank	2017–10–31	Nigeria	Human	IIb	A	phylogeny
OP535322.1	GenBank	2017–10–31	Nigeria	Human	IIb	A	phylogeny
OP535323.1	GenBank	2017–10–01	Nigeria	Human	IIb	A.3	phylogeny
OP535324.1	GenBank	2018–02–01	Nigeria	Human	IIb	A	phylogeny
OP535325.1	GenBank	2018–06–01	Nigeria	Human	IIb	A	phylogeny
OP535326.1	GenBank	2018–06–01	Nigeria	Human	IIb	A	phylogeny
OP535327.1	GenBank	2018–03–02	Nigeria	Human	IIb	A	phylogeny
OP535328.1	GenBank	2018–03–01	Nigeria	Human	IIb	A	phylogeny
OP535329.1	GenBank	2018–03–01	Nigeria	Human	IIb	A	phylogeny
OP535330.1	GenBank	2018–02–01	Nigeria	Human	IIb	A	phylogeny
OP535331.1	GenBank	2018–01–01	Nigeria	Human	IIb	A	phylogeny
OP535332.1	GenBank	2018–01–01	Nigeria	Human	IIb	A	phylogeny
OP535333.1	GenBank	2018–01–31	Nigeria	Human	IIb	A	phylogeny
OP535334.1	GenBank	2018–02–01	Nigeria	Human	IIb	A	phylogeny
OP535335.1	GenBank	2017–10–01	Nigeria	Human	IIb	A.3	phylogeny

Accession number	Source	Date of collection	Country	Host	Clade	Lineage	Analysis
OP535336.1	GenBank	2017–10–01	Nigeria	Human	IIb	A	phylogeny
OP535337.1	GenBank	2017–10–31	Nigeria	Human	IIb	A	phylogeny
OP535338.1	GenBank	2017–10–31	Nigeria	Human	IIb	A	phylogeny
OP535339.1	GenBank	2017–10–01	Nigeria	Human	IIb	A	phylogeny
OP535340.1	GenBank	2017–10–31	Nigeria	Human	IIb	A	phylogeny
OP535341.1	GenBank	2017–10–01	Nigeria	Human	IIb	A	phylogeny
OP959058.1	GenBank	2022–10–27	Colombia	Human	IIb	B.1.2	phylogeny
OP936000.1	GenBank	2022–09–26	Vietnam	Human	IIb	A.2.1	phylogeny
OP936001.1	GenBank	2022–10–19	Vietnam	Human	IIb	A.2.1	phylogeny
OP838893.1	GenBank	2022–06–21	Italy	Human	IIb	B.1.19	phylogeny
OP605560.1	GenBank	2022–07–31	Slovenia	Human	IIb	B.1	phylogeny
OP605572.1	GenBank	2022–07–01	Slovenia	Human	IIb	A.2.1	phylogeny
OP612674.1	GenBank	2019–01–12	Nigeria	Human	IIb	A.1	phylogeny
OP612675.1	GenBank	2019–01–30	Nigeria	Human	IIb	A	phylogeny
OP612677.1	GenBank	2019–03–12	Nigeria	Human	IIb	A.3	phylogeny
OP612678.1	GenBank	2019–03–14	Nigeria	Human	IIb	A	phylogeny
OP612679.1	GenBank	2019–03–14	Nigeria	Human	IIb	A.1	phylogeny
OP612680.1	GenBank	2019–04–01	Nigeria	Human	IIb	A	phylogeny
OP612681.1	GenBank	2019–04–20	Nigeria	Human	IIb	A.1	phylogeny
OP612682.1	GenBank	2019–04–20	Nigeria	Human	IIb	A.1	phylogeny
OP612683.1	GenBank	2019–04–18	Nigeria	Human	IIb	A.1	phylogeny
OP612684.1	GenBank	2019–05–01	Nigeria	Human	IIb	A	phylogeny
OP612685.1	GenBank	2019–05–29	Nigeria	Human	IIb	A.1	phylogeny
OP612686.1	GenBank	2019–06–26	Nigeria	Human	IIb	A.1	phylogeny
OP612687.1	GenBank	2019–07–11	Nigeria	Human	IIb	A.1	phylogeny
OP612688.1	GenBank	2019–11–02	Nigeria	Human	IIb	A.1	phylogeny
OP612689.1	GenBank	2019–12–17	Nigeria	Human	IIb	A.2	phylogeny
OP612690.1	GenBank	2019–12–17	Nigeria	Human	IIb	A.2	phylogeny
OP612691.1	GenBank	2020–01–31	Nigeria	Human	IIb	A.2	phylogeny
OP597769.1	GenBank	2022–09–26	Egypt	Human	IIb	A.2.1	phylogeny
OX344884.1	GenBank	2022–05–24	Spain	Human	IIb	B.1	phylogeny
OX344887.1	GenBank	2022–04–27	Spain	Human	IIb	B.1	phylogeny
OP555485.1	GenBank	2022–07–12	Portugal	Human	IIb	B.1.9	phylogeny
OP555515.1	GenBank	2022–08–01	Portugal	Human	IIb	A.2.3	phylogeny
OP555657.1	GenBank	2022–08–24	Portugal	Human	IIb	B.1	phylogeny
OP536812.1	GenBank	2022–09–01	United Kingdom	Human	IIb	A.3	phylogeny
OP450997.1	GenBank	2022–07–01	USA	Human	IIb	A.2.2	phylogeny
OX336038.1	GenBank	2022–08–31	Slovakia	Human	IIb	B.1	phylogeny
OP422340.1	GenBank	2022–07–13	Austria	Human	IIb	B.1	phylogeny
OP413718.1	GenBank	2022–08–31	United Kingdom	Human	IIb	A.3	phylogeny
OP415232.1	GenBank	2022–08–01	United Kingdom	Human	IIb	B.1.2	phylogeny
OP415283.1	GenBank	2022–08–04	United Kingdom	Human	IIb	B.1	phylogeny
OP382485.1	GenBank	2022–07–05	France	Human	IIb	B.1.12	phylogeny
OP382492.1	GenBank	2022–07–22	France	Human	IIb	B.1.14	phylogeny
OP331335.1	GenBank	2022–06–03	United Kingdom	Human	IIb	A.2.1	phylogeny
OP331336.1	GenBank	2022–06–29	United Kingdom	Human	IIb	A.2.2	phylogeny
OP320548.1	GenBank	2022–07–01	Peru	Human	IIb	B.1.6	phylogeny
OP320554.1	GenBank	2022–07–09	Peru	Human	IIb	B.1.6	phylogeny
OX291694.1	GenBank	2022–08–01	Slovakia	Human	IIb	B.1	phylogeny
OP204857.1	GenBank	2022–08–01	South Korea	Human	IIb	B.1.1	phylogeny
OP160532.1	GenBank	2022–06–01	Netherlands	Human	IIb	B.1	phylogeny
OP144215.2	GenBank	2022–05–30	Belgium	Human	IIb	B.1	phylogeny
OP133007.1	GenBank	2022–07–22	Austria	Human	IIb	B.1	phylogeny
ON918611.1	GenBank	2022–06–20	South Africa	Human	IIb	B.1.7	phylogeny
ON927248.1	GenBank	2022–06–24	South Africa	Human	IIb	B.1.7	phylogeny
ON983168.1	GenBank	2022–04–28	Czech Republic	Human	IIb	B.1.3	phylogeny
ON959143.1	GenBank	2022–06–16	Finland	Human	IIb	B.1.10	phylogeny
OL504741.1	GenBank	2019–12–15	United Kingdom	Human	IIb	A.1	phylogeny
OL504742.1	GenBank	2019–12–15	United Kingdom	Human	IIb	A.1	phylogeny

Accession number	Source	Date of collection	Country	Host	Clade	Lineage	Analysis
OL504743.1	GenBank	2019–12–16	United Kingdom	Human	IIb	A.1	phylogeny
ON918656.1	GenBank	2022–06–30	Taiwan	Human	IIb	B.1.5	phylogeny
ON911481.2	GenBank	2022–06–28	Mexico	Human	IIb	B.1	phylogeny
ON880413.1	GenBank	2022–06–14	Brazil	Human	IIb	B.1.12	phylogeny
ON880530.1	GenBank	2022–05–29	Canada	Human	IIb	B.1	phylogeny
ON880531.1	GenBank	2022–05–29	Canada	Human	IIb	B.1	phylogeny
ON872184.1	GenBank	2022–05–30	Ireland	Human	IIb	B.1	phylogeny
ON782022.1	GenBank	2022–05–31	Finland	Human	IIb	B.1	phylogeny
ON745215.1	GenBank	2022–05–19	Italy	Human	IIb	B.1	phylogeny
ON751962.1	GenBank	2022–06–07	Brazil	Human	IIb	B.1	phylogeny
ON674051.1	GenBank	2022–05–01	USA	Human	IIb	A.2.1	phylogeny
ON675438.1	GenBank	2022–05–01	USA	Human	IIb	A.2	phylogeny
ON676707.1	GenBank	2021–07–31	USA	Human	IIb	A.2.3	phylogeny
ON676708.1	GenBank	2021–11–01	USA	Human	IIb	A.1.1	phylogeny
ON649879.1	GenBank	2022–05–20	Israel	Human	IIb	B.1	phylogeny
OX009124.1	GenBank	2022–05–22	Sweden	Human	IIb	B.1.14	phylogeny
ON622720.2	GenBank	2022–05–24	Switzerland	Human	IIb	B.1	phylogeny
ON615424.1	GenBank	2022–05–31	Netherlands	Human	IIb	B.1	phylogeny
MT903337.1	GenBank	2018–01–01	Nigeria	Human	IIb	A	phylogeny
MT903338.1	GenBank	2018–01–01	Nigeria	Human	IIb	A	phylogeny
MT903339.1	GenBank	2018–01–01	Nigeria	Human	IIb	A	phylogeny
MT903341.1	GenBank	2018–08–14	Nigeria	Human	IIb	A.1	phylogeny
MT903342.1	GenBank	2019–05–30	Singapore	Human	IIb	A.1	phylogeny
MT903343.1	GenBank	2018–09–30	United Kingdom	Human	IIb	A.1	phylogeny
MT903344.1	GenBank	2018–09–22	United Kingdom	Human	IIb	A.1	phylogeny
MT903345.1	GenBank	2018–09–22	United Kingdom	Human	IIb	A.1	phylogeny
MT250197.1	GenBank	2019–06–07	Singapore	Human	IIb	A.1	phylogeny
MN648051.1	GenBank	2018–10–04	Israel	Human	IIb	A.1	phylogeny
MK783027.1	GenBank	2017–11–09	Nigeria	Human	IIb	A	phylogeny
MK783028.1	GenBank	2017–11–09	Nigeria	Human	IIb	A	phylogeny
MK783029.1	GenBank	2017–12–06	Nigeria	Human	IIb	A	phylogeny
MK783030.1	GenBank	2017–11–30	Nigeria	Human	IIb	A	phylogeny
MK783031.1	GenBank	2017–11–09	Nigeria	Human	IIb	A	phylogeny
MK783032.1	GenBank	2017–11–01	Nigeria	Human	IIb	A	phylogeny
MK783033.1	GenBank	2017–10–09	Nigeria	Human	IIb	A.3	phylogeny
MG693723.1	GenBank	2017–12–31	Nigeria	Human	IIb	A.3	phylogeny
MG693724.1	GenBank	2017–12–31	Nigeria	Human	IIb	A	phylogeny
EPI_ISL_19108154	GISAID	2023–10–02	Vietnam	Human	IIb	C.1	phylogeny&intra-host mutation
EPI_ISL_19108159	GISAID	2023–10–02	Vietnam	Human	IIb	C.1	phylogeny
EPI_ISL_19108160	GISAID	2023–10–02	Vietnam	Human	IIb	C.1	phylogeny
EPI_ISL_19108161	GISAID	2023–10–04	Vietnam	Human	IIb	C.1	phylogeny
EPI_ISL_19108162	GISAID	2023–10–05	Vietnam	Human	IIb	C.1	phylogeny
EPI_ISL_19108163	GISAID	2023–10–09	Vietnam	Human	IIb	C.1	phylogeny
EPI_ISL_19108164	GISAID	2023–10–10	Vietnam	Human	IIb	C.1	phylogeny
EPI_ISL_19108165	GISAID	2023–10–11	Vietnam	Human	IIb	C.1	phylogeny
EPI_ISL_19108166	GISAID	2023–10–11	Vietnam	Human	IIb	C.1	phylogeny
EPI_ISL_19108167	GISAID	2023–10–12	Vietnam	Human	IIb	C.1	phylogeny
EPI_ISL_19108168	GISAID	2023–10–13	Vietnam	Human	IIb	C.1	phylogeny
EPI_ISL_19108169	GISAID	2023–10–14	Vietnam	Human	IIb	C.1	phylogeny
EPI_ISL_19108170	GISAID	2023–10–16	Vietnam	Human	IIb	C.1	phylogeny
EPI_ISL_18993959	GISAID	2024–01–15	Portugal	Human	IIb	C.1	phylogeny
EPI_ISL_18993966	GISAID	2024–01–13	Portugal	Human	IIb	C.1	phylogeny
EPI_ISL_18993967	GISAID	2023–12–21	Portugal	Human	IIb	C.1	phylogeny
EPI_ISL_18993961	GISAID	2023–12–27	Portugal	Human	IIb	C.1	phylogeny
EPI_ISL_18993964	GISAID	2023–11–30	Portugal	Human	IIb	C.1	phylogeny
EPI_ISL_18702237	GISAID	2023–10–13	Portugal	Human	IIb	C.1	phylogeny
EPI_ISL_18702236	GISAID	2023–09–22	Portugal	Human	IIb	C.1	phylogeny
EPI_ISL_18076384	GISAID	2023–07–10	Portugal	Human	IIb	C.1	phylogeny
EPI_ISL_18702243	GISAID	2023–10–27	Portugal	Human	IIb	C.1	phylogeny
EPI_ISL_18228634	GISAID	2023–08–02	Portugal	Human	IIb	C.1	phylogeny
EPI_ISL_18553811	GISAID	2023–09–19	Portugal	Human	IIb	C.1	phylogeny
EPI_ISL_18553850	GISAID	2023–08–21	Portugal	Human	IIb	C.1	phylogeny
EPI_ISL_18228630	GISAID	2023–08–01	Portugal	Human	IIb	C.1	phylogeny

Accession number	Source	Date of collection	Country	Host	Clade	Lineage	Analysis
EPI_ISL_18659829	GISAID	2023–10–15	Netherlands	Human	IIb	C.1	phylogeny
EPI_ISL_18971017	GISAID	2023–11–08	Brazil	Human	IIb	C.1	phylogeny
EPI_ISL_18659828	GISAID	2023–08–29	Netherlands	Human	IIb	C.1	phylogeny
EPI_ISL_18746884	GISAID	2023–12–18	Netherlands	Human	IIb	C.1	phylogeny
EPI_ISL_18553829	GISAID	2023–08–07	Portugal	Human	IIb	C.1	phylogeny
EPI_ISL_18228645	GISAID	2023–07–21	Portugal	Human	IIb	C.1	phylogeny
EPI_ISL_18689511	GISAID	2023–11–22	Germany	Human	IIb	C.1	phylogeny
EPI_ISL_18228631	GISAID	2023–07–21	Portugal	Human	IIb	C.1	phylogeny
EPI_ISL_18213375	GISAID	2023–06–19	People's Republic of China	Human	IIb	C.1	phylogeny
EPI_ISL_18213374	GISAID	2023–06–24	People's Republic of China	Human	IIb	C.1	phylogeny
EPI_ISL_18702235	GISAID	2023–10–02	Portugal	Human	IIb	C.1	phylogeny
EPI_ISL_19001887	GISAID	2024–01–25	Japan	Human	IIb	C.1	phylogeny
EPI_ISL_17809521	GISAID	2023–06–15	People's Republic of China	Human	IIb	C.1	phylogeny
EPI_ISL_18075506	GISAID	2023–05–01	Japan	Human	IIb	C.1	phylogeny
EPI_ISL_17665624	GISAID	2023–04–17	Japan	Human	IIb	C.1	phylogeny
EPI_ISL_18097375	GISAID	2023–05–02	Japan	Human	IIb	C.1	phylogeny
EPI_ISL_17817240	GISAID	2023–05–19	Japan	Human	IIb	C.1	phylogeny
EPI_ISL_17592667	GISAID	2023–04–06	Japan	Human	IIb	C.1	phylogeny
EPI_ISL_18075508	GISAID	2023–07–07	Japan	Human	IIb	C.1	phylogeny
EPI_ISL_18739595	GISAID	2023–12–01	Japan	Human	IIb	C.1	phylogeny
EPI_ISL_18414668	GISAID	2023–09	USA	Human	IIb	C.1	phylogeny
EPI_ISL_18125040	GISAID	2023–06	USA	Human	IIb	C.1	phylogeny
EPI_ISL_19002300	GISAID	2024–03–07	Netherlands	Human	IIb	C.1	phylogeny
EPI_ISL_18147347	GISAID	2023–04–22	South Korea	Human	IIb	C.1	phylogeny
EPI_ISL_18744050	GISAID	2023–05–03	South Korea	Human	IIb	C.1	phylogeny
EPI_ISL_17592666	GISAID	2023–04–04	Japan	Human	IIb	C.1	phylogeny
EPI_ISL_17445518	GISAID	2023–03–31	Japan	Human	IIb	C.1	phylogeny
EPI_ISL_18147350	GISAID	2023–04–25	South Korea	Human	IIb	C.1	phylogeny
EPI_ISL_16905444	GISAID	2022–10	Japan	Human	IIb	C.1	phylogeny
EPI_ISL_17152843	GISAID	2023–02	Taiwan	Human	IIb	C.1	phylogeny
EPI_ISL_17428286	GISAID	2023–03	Taiwan	Human	IIb	C.1	phylogeny
EPI_ISL_17152764	GISAID	2023–03	Taiwan	Human	IIb	C.1	phylogeny
EPI_ISL_18147336	GISAID	2023–04–22	South Korea	Human	IIb	C.1	phylogeny
EPI_ISL_18147340	GISAID	2023–04–14	South Korea	Human	IIb	C.1	phylogeny
EPI_ISL_18147343	GISAID	2023–04–21	South Korea	Human	IIb	C.1	phylogeny
EPI_ISL_18147353	GISAID	2023–04–21	South Korea	Human	IIb	C.1	phylogeny
EPI_ISL_18147361	GISAID	2023–04–17	South Korea	Human	IIb	C.1	phylogeny
EPI_ISL_18147358	GISAID	2023–04–18	South Korea	Human	IIb	C.1	phylogeny
EPI_ISL_18147334	GISAID	2023–04–13	South Korea	Human	IIb	C.1	phylogeny
EPI_ISL_18147360	GISAID	2023–04–11	South Korea	Human	IIb	C.1	phylogeny
EPI_ISL_18147344	GISAID	2023–04–19	South Korea	Human	IIb	C.1	phylogeny
EPI_ISL_18147363	GISAID	2023–04–18	South Korea	Human	IIb	C.1	phylogeny
EPI_ISL_18436040	GISAID	2023–10–12	Indonesia	Human	IIb	C.1	phylogeny
EPI_ISL_18467807	GISAID	2023–10–30	Indonesia	Human	IIb	C.1	phylogeny
EPI_ISL_18467808	GISAID	2023–10–25	Indonesia	Human	IIb	C.1	phylogeny
EPI_ISL_18467797	GISAID	2023–10–27	Indonesia	Human	IIb	C.1	phylogeny
EPI_ISL_18467796	GISAID	2023–10–23	Indonesia	Human	IIb	C.1	phylogeny
EPI_ISL_18467794	GISAID	2023–10–27	Indonesia	Human	IIb	C.1	phylogeny
EPI_ISL_18463161	GISAID	2023–10–21	Indonesia	Human	IIb	C.1	phylogeny
EPI_ISL_18467799	GISAID	2023–10–23	Indonesia	Human	IIb	C.1	phylogeny
EPI_ISL_18467798	GISAID	2023–10–27	Indonesia	Human	IIb	C.1	phylogeny
EPI_ISL_18467801	GISAID	2023–10–25	Indonesia	Human	IIb	C.1	phylogeny
EPI_ISL_18436041	GISAID	2023–10–20	Indonesia	Human	IIb	C.1	phylogeny
EPI_ISL_17246659	GISAID	2023–02–13	Japan	Human	IIb	C.1	phylogeny
EPI_ISL_17428283	GISAID	2023–03	Taiwan	Human	IIb	C.1	phylogeny
EPI_ISL_17445519	GISAID	2023–03–31	Japan	Human	IIb	C.1	phylogeny
EPI_ISL_17692268	GISAID	2023–04–28	Japan	Human	IIb	C.1	phylogeny
EPI_ISL_17837267	GISAID	2023–06–12	Japan	Human	IIb	C.1	phylogeny
EPI_ISL_17959214	GISAID	2023–05–11	Japan	Human	IIb	C.1	phylogeny
EPI_ISL_17518107	GISAID	2023–03–17	Japan	Human	IIb	C.1	phylogeny
EPI_ISL_17762485	GISAID	2023–05–17	Japan	Human	IIb	C.1	phylogeny
EPI_ISL_17959215	GISAID	2023–05–11	Japan	Human	IIb	C.1	phylogeny

Accession number	Source	Date of collection	Country	Host	Clade	Lineage	Analysis
EPI_ISL_17959216	GISAID	2023–05–12	Japan	Human	IIb	C.1	phylogeny
EPI_ISL_17665625	GISAID	2023–04–18	Japan	Human	IIb	C.1	phylogeny
EPI_ISL_17592665	GISAID	2023–04–03	Japan	Human	IIb	C.1	phylogeny
EPI_ISL_17008295	GISAID	2023–01–30	Japan	Human	IIb	C.1	phylogeny
EPI_ISL_18147345	GISAID	2023–04–10	South Korea	Human	IIb	C.1	phylogeny
EPI_ISL_18147348	GISAID	2023–04–18	South Korea	Human	IIb	C.1	phylogeny
EPI_ISL_18147349	GISAID	2023–04–22	South Korea	Human	IIb	C.1	phylogeny
EPI_ISL_17008296	GISAID	2023–02–09	Japan	Human	IIb	C.1	phylogeny
EPI_ISL_18147342	GISAID	2023–04–15	South Korea	Human	IIb	C.1	phylogeny
EPI_ISL_17837266	GISAID	2023–06–02	Japan	Human	IIb	C.1	phylogeny
EPI_ISL_17445516	GISAID	2023–03–28	Japan	Human	IIb	C.1	phylogeny
EPI_ISL_18352304	GISAID	2023–09–14	Japan	Human	IIb	C.1	phylogeny
EPI_ISL_18147346	GISAID	2023–04–25	South Korea	Human	IIb	C.1	phylogeny
EPI_ISL_17008293	GISAID	2023–01–25	Japan	Human	IIb	C.1	phylogeny
EPI_ISL_17592670	GISAID	2023–04–11	Japan	Human	IIb	C.1	phylogeny
EPI_ISL_17665626	GISAID	2023–04–19	Japan	Human	IIb	C.1	phylogeny
EPI_ISL_17246657	GISAID	2023–01–23	Japan	Human	IIb	C.1	phylogeny
EPI_ISL_18147351	GISAID	2023–04–21	South Korea	Human	IIb	C.1	phylogeny
EPI_ISL_18697752	GISAID	2023–12–11	Cambodia	Human	IIb	C.1	phylogeny
EPI_ISL_18147359	GISAID	2023–04–12	South Korea	Human	IIb	C.1	phylogeny
EPI_ISL_18055899	GISAID	2023–06–26	Japan	Human	IIb	C.1	phylogeny
EPI_ISL_18147355	GISAID	2023–03–13	South Korea	Human	IIb	C.1	phylogeny
EPI_ISL_18557816	GISAID	2023–09–22	Japan	Human	IIb	C.1	phylogeny
EPI_ISL_18557818	GISAID	2023–10–25	Japan	Human	IIb	C.1	phylogeny
EPI_ISL_18075507	GISAID	2023–05–01	Japan	Human	IIb	C.1	phylogeny
EPI_ISL_17817241	GISAID	2023–05–20	Japan	Human	IIb	C.1	phylogeny
EPI_ISL_17817239	GISAID	2023–05–15	Japan	Human	IIb	C.1	phylogeny
EPI_ISL_17837268	GISAID	2023–06–09	Japan	Human	IIb	C.1	phylogeny
EPI_ISL_17665627	GISAID	2023–04–21	Japan	Human	IIb	C.1	phylogeny
EPI_ISL_18055900	GISAID	2023–06–26	Japan	Human	IIb	C.1	phylogeny
EPI_ISL_17692269	GISAID	2023–04–28	Japan	Human	IIb	C.1	phylogeny
EPI_ISL_18352303	GISAID	2023–09–11	Japan	Human	IIb	C.1	phylogeny
EPI_ISL_18352306	GISAID	2023–09–19	Japan	Human	IIb	C.1	phylogeny
EPI_ISL_18059184	GISAID	2023–07–03	People’s Republic of China	Human	IIb	C.1	phylogeny
EPI_ISL_18659846	GISAID	2023–11–07	Netherlands	Human	IIb	C.1	phylogeny
EPI_ISL_18702225	GISAID	2023–10–06	Portugal	Human	IIb	C.1	phylogeny
EPI_ISL_18702257	GISAID	2023–10–31	Portugal	Human	IIb	C.1	phylogeny
EPI_ISL_18059183	GISAID	2023–06–28	People’s Republic of China	Human	IIb	C.1	phylogeny
EPI_ISL_18059182	GISAID	2023–06–27	People’s Republic of China	Human	IIb	C.1	phylogeny
EPI_ISL_18076380	GISAID	2023–07–07	Portugal	Human	IIb	C.1	phylogeny
EPI_ISL_18553846	GISAID	2023–08–21	Portugal	Human	IIb	C.1	phylogeny
EPI_ISL_18553847	GISAID	2023–08–08	Portugal	Human	IIb	C.1	phylogeny
EPI_ISL_18702208	GISAID	2023–10–17	Portugal	Human	IIb	C.1	phylogeny
EPI_ISL_18702253	GISAID	2023–10–19	Portugal	Human	IIb	C.1	phylogeny
EPI_ISL_18702234	GISAID	2023–10–29	Portugal	Human	IIb	C.1	phylogeny
EPI_ISL_18044983	GISAID	2023–06–20	Portugal	Human	IIb	C.1	phylogeny
EPI_ISL_18702241	GISAID	2023–10–03	Portugal	Human	IIb	C.1	phylogeny
EPI_ISL_18553835	GISAID	2023–07–18	Portugal	Human	IIb	C.1	phylogeny
EPI_ISL_18553816	GISAID	2023–07–13	Portugal	Human	IIb	C.1	phylogeny
EPI_ISL_18076389	GISAID	2023–07–07	Portugal	Human	IIb	C.1	phylogeny
EPI_ISL_18553833	GISAID	2023–08–10	Portugal	Human	IIb	C.1	phylogeny
EPI_ISL_18228633	GISAID	2023–07–19	Portugal	Human	IIb	C.1	phylogeny
EPI_ISL_18634755	GISAID	2023–10	Ireland	Human	IIb	C.1	phylogeny
EPI_ISL_18228640	GISAID	2023–07–26	Portugal	Human	IIb	C.1	phylogeny
EPI_ISL_18746885	GISAID	2023–12–19	Netherlands	Human	IIb	C.1	phylogeny
EPI_ISL_18781817	GISAID	2023–10–14	USA	Human	IIb	C.1	phylogeny
EPI_ISL_18634756	GISAID	2023–10	Ireland	Human	IIb	C.1	phylogeny
EPI_ISL_18308397	GISAID	2023–08–18	Ireland	Human	IIb	C.1	phylogeny
EPI_ISL_18308399	GISAID	2023–08–28	Ireland	Human	IIb	C.1	phylogeny
EPI_ISL_18308398	GISAID	2023–08–31	Ireland	Human	IIb	C.1	phylogeny
EPI_ISL_18308396	GISAID	2023–08–16	Ireland	Human	IIb	C.1	phylogeny
EPI_ISL_18553837	GISAID	2023–07–15	Portugal	Human	IIb	C.1	phylogeny

Accession number	Source	Date of collection	Country	Host	Clade	Lineage	Analysis
EPI_ISL_18228621	GISAID	2023–07–26	Portugal	Human	IIb	C.1	phylogeny
EPI_ISL_18702249	GISAID	2023–10–20	Portugal	Human	IIb	C.1	phylogeny
EPI_ISL_18702212	GISAID	2023–09–24	Portugal	Human	IIb	C.1	phylogeny
EPI_ISL_18702252	GISAID	2023–11–20	Portugal	Human	IIb	C.1	phylogeny
EPI_ISL_18702229	GISAID	2023–10–29	Portugal	Human	IIb	C.1	phylogeny
EPI_ISL_18702224	GISAID	2023–10–12	Portugal	Human	IIb	C.1	phylogeny
EPI_ISL_18702227	GISAID	2023–11–09	Portugal	Human	IIb	C.1	phylogeny
EPI_ISL_18702256	GISAID	2023–10–12	Portugal	Human	IIb	C.1	phylogeny
EPI_ISL_18702223	GISAID	2023–11–02	Portugal	Human	IIb	C.1	phylogeny
EPI_ISL_18553813	GISAID	2023–09–08	Portugal	Human	IIb	C.1	phylogeny
EPI_ISL_18702255	GISAID	2023–09–19	Portugal	Human	IIb	C.1	phylogeny
EPI_ISL_18702250	GISAID	2023–09–22	Portugal	Human	IIb	C.1	phylogeny
EPI_ISL_18702231	GISAID	2023–09–26	Portugal	Human	IIb	C.1	phylogeny
EPI_ISL_18702221	GISAID	2023–11–07	Portugal	Human	IIb	C.1	phylogeny
EPI_ISL_18702230	GISAID	2023–09–29	Portugal	Human	IIb	C.1	phylogeny
EPI_ISL_18702240	GISAID	2023–11–01	Portugal	Human	IIb	C.1	phylogeny
EPI_ISL_18553826	GISAID	2023–09–12	Portugal	Human	IIb	C.1	phylogeny
EPI_ISL_18702226	GISAID	2023–10–18	Portugal	Human	IIb	C.1	phylogeny
EPI_ISL_18702247	GISAID	2023–10–03	Portugal	Human	IIb	C.1	phylogeny
EPI_ISL_18702209	GISAID	2023–11–18	Portugal	Human	IIb	C.1	phylogeny
EPI_ISL_18702251	GISAID	2023–10–26	Portugal	Human	IIb	C.1	phylogeny
OR459803.1	GenBank	2023–04–25	South Korea	Human	IIb	C.1	phylogeny
OR459792.1	GenBank	2023–04–19	South Korea	Human	IIb	C.1	phylogeny
OR459799.1	GenBank	2023–04–22	South Korea	Human	IIb	C.1	phylogeny
OR459784.1	GenBank	2023–04–14	South Korea	Human	IIb	C.1	phylogeny
OR459794.1	GenBank	2023–04–21	South Korea	Human	IIb	C.1	phylogeny
OR459790.1	GenBank	2023–04–18	South Korea	Human	IIb	C.1	phylogeny
OR459796.1	GenBank	2023–04–21	South Korea	Human	IIb	C.1	phylogeny
OR459782.1	GenBank	2023–04–12	South Korea	Human	IIb	C.1	phylogeny
OR427348.1	GenBank	2023–06	USA	Human	IIb	C.1	phylogeny
OR459779.1	GenBank	2023–04–06	South Korea	Human	IIb	C.1	phylogeny
OR459804.1	GenBank	2023–04–25	South Korea	Human	IIb	C.1	phylogeny
OR459785.1	GenBank	2023–04–15	South Korea	Human	IIb	C.1	phylogeny
OR459780.1	GenBank	2023–04–10	South Korea	Human	IIb	C.1	phylogeny
OR459791.1	GenBank	2023–04–18	South Korea	Human	IIb	C.1	phylogeny
OR459797.1	GenBank	2023–04–22	South Korea	Human	IIb	C.1	phylogeny
OR459798.1	GenBank	2023–04–22	South Korea	Human	IIb	C.1	phylogeny
OR459788.1	GenBank	2023–04–18	South Korea	Human	IIb	C.1	phylogeny
OR459781.1	GenBank	2023–04–11	South Korea	Human	IIb	C.1	phylogeny
OR665166.1	GenBank	2023–09	USA	Human	IIb	C.1	phylogeny
OR459778.1	GenBank	2023–03–13	South Korea	Human	IIb	C.1	phylogeny
OR972728.1	GenBank	2023–11–22	Germany	Human	IIb	C.1	phylogeny
OR855754.1	GenBank	2023–07–05	South Korea	Human	IIb	C.1	phylogeny
OR855745.1	GenBank	2023–06–01	South Korea	Human	IIb	C.1	phylogeny
OR855755.1	GenBank	2023–07–05	South Korea	Human	IIb	C.1	phylogeny
OR855753.1	GenBank	2023–09–15	South Korea	Human	IIb	C.1	phylogeny
OR855752.1	GenBank	2023–07–05	South Korea	Human	IIb	C.1	phylogeny
OR855765.1	GenBank	2023–08–03	South Korea	Human	IIb	C.1	phylogeny
OR855758.1	GenBank	2023–07–05	South Korea	Human	IIb	C.1	phylogeny
OR855757.1	GenBank	2023–07–05	South Korea	Human	IIb	C.1	phylogeny
OR855756.1	GenBank	2023–07–05	South Korea	Human	IIb	C.1	phylogeny
OR855761.1	GenBank	2023–09–15	South Korea	Human	IIb	C.1	phylogeny
OR855759.1	GenBank	2023–09–15	South Korea	Human	IIb	C.1	phylogeny
OR855751.1	GenBank	2023–07–05	South Korea	Human	IIb	C.1	phylogeny
OR855746.1	GenBank	2023–06–23	South Korea	Human	IIb	C.1	phylogeny
OR855748.1	GenBank	2023–06–23	South Korea	Human	IIb	C.1	phylogeny
OR855766.1	GenBank	2023–08–28	South Korea	Human	IIb	C.1	phylogeny
OR855767.1	GenBank	2023–08–28	South Korea	Human	IIb	C.1	phylogeny
OR855749.1	GenBank	2023–07–05	South Korea	Human	IIb	C.1	phylogeny
OR459795.1	GenBank	2023–04–21	South Korea	Human	IIb	C.1	phylogeny
OR855750.1	GenBank	2023–07–05	South Korea	Human	IIb	C.1	phylogeny
OR855744.1	GenBank	2023–05–03	South Korea	Human	IIb	C.1	phylogeny
OR459787.1	GenBank	2023–04–17	South Korea	Human	IIb	C.1	phylogeny
OR487991.1	GenBank	2023–08–02	Portugal	Human	IIb	C.1	phylogeny
OR487992.1	GenBank	2023–08–02	Portugal	Human	IIb	C.1	phylogeny
OR459783.1	GenBank	2023–04–13	South Korea	Human	IIb	C.1	phylogeny
OR459802.1	GenBank	2023–04–25	South Korea	Human	IIb	C.1	phylogeny
LC760460.1	GenBank	2023–01–23	Japan	Human	IIb	C.1	phylogeny

Accession number	Source	Date of collection	Country	Host	Clade	Lineage	Analysis
LC756923.1	GenBank	2023–01–25	Japan	Human	IIb	C.1	phylogeny
LC753968.1	GenBank	2022–09–30	Japan	Human	IIb	C.1	phylogeny
LC753969.1	GenBank	2022–10–01	Japan	Human	IIb	C.1	phylogeny
EPI_ISL_19108171	GISAID	2023–09–24	Vietnam	Human	IIb	C.1	phylogeny
OR326991.1	GenBank	2023–06–15	Portugal	Human	IIb	C.1	phylogeny
OR326992.1	GenBank	2023–06–16	Portugal	Human	IIb	C.1	phylogeny
OR326993.1	GenBank	2023–06–20	Portugal	Human	IIb	C.1	phylogeny
OR326994.1	GenBank	2023–06–22	Portugal	Human	IIb	C.1	phylogeny
OR326995.1	GenBank	2023–06–22	Portugal	Human	IIb	C.1	phylogeny
OR326996.1	GenBank	2023–06–25	Portugal	Human	IIb	C.1	phylogeny
OR326997.1	GenBank	2023–06–25	Portugal	Human	IIb	C.1	phylogeny
OR035728.1	GenBank	2022–10–28	Belgium	Human	IIb	C.1	phylogeny
OQ721952.1	GenBank	2023–03	Taiwan	Human	IIb	C.1	phylogeny
LC760461.1	GenBank	2023–02–13	Japan	Human	IIb	C.1	phylogeny
OQ581853.1	GenBank	2023–02	Taiwan	Human	IIb	C.1	phylogeny
OQ581855.1	GenBank	2023–03	Taiwan	Human	IIb	C.1	phylogeny
LC756922.1	GenBank	2023–01–25	Japan	Human	IIb	C.1	phylogeny
LC756924.1	GenBank	2023–01–30	Japan	Human	IIb	C.1	phylogeny
LC756925.1	GenBank	2023–02–09	Japan	Human	IIb	C.1	phylogeny
EPI_ISL_19108155	GISAID	2023–10–09	Vietnam	Human	IIb	C.1	intra-host mutation
EPI_ISL_19108156	GISAID	2023–10–16	Vietnam	Human	IIb	C.1	intra-host mutation
EPI_ISL_19108157	GISAID	2023–10–11	Vietnam	Human	IIb	C.1	intra-host mutation
EPI_ISL_19108158	GISAID	2023–10–19	Vietnam	Human	IIb	C.1	intra-host mutation

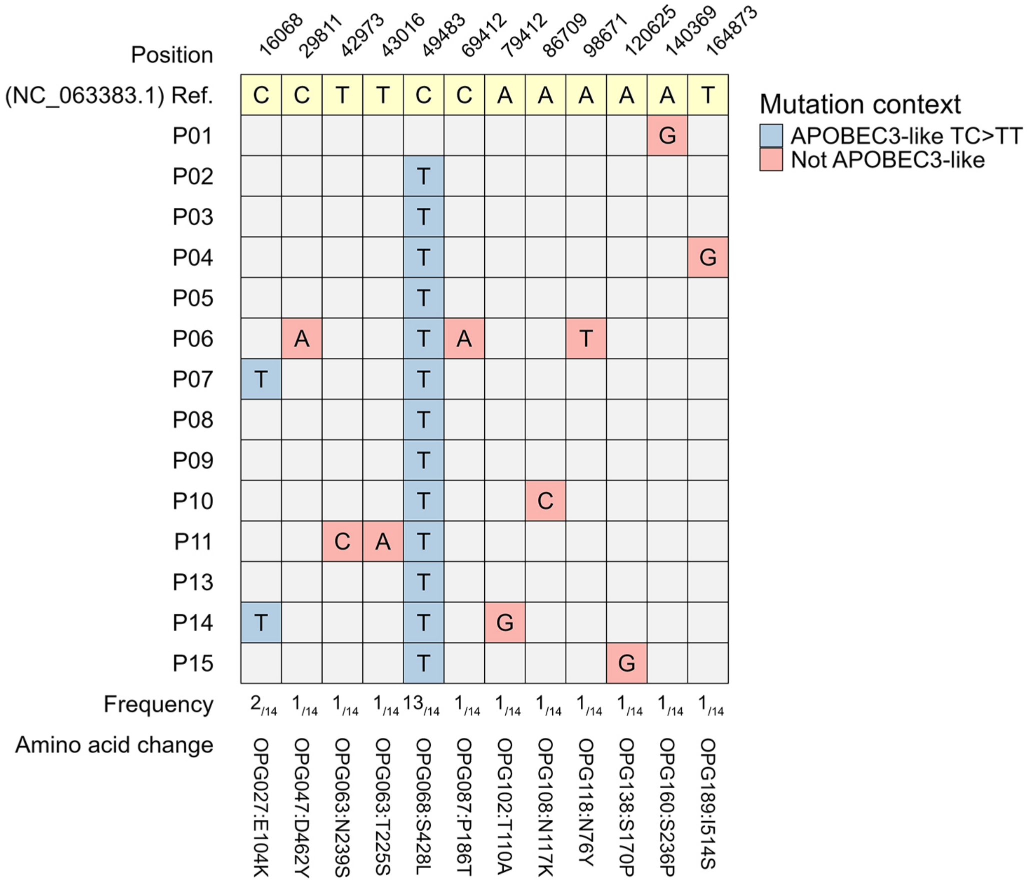

Appendix Figure 1. Novel nonsynonymous substitutions documents in 14 sequences of the present study. Notes to Appendix Figure 1: E: Glutamate, K: Lysine, D: Aspartate, Y: Tyrosine, S: Serine, N: Asparagine, T: Threonine, L: Leucine, P: Proline, A: Alanine and I: Isoleucine

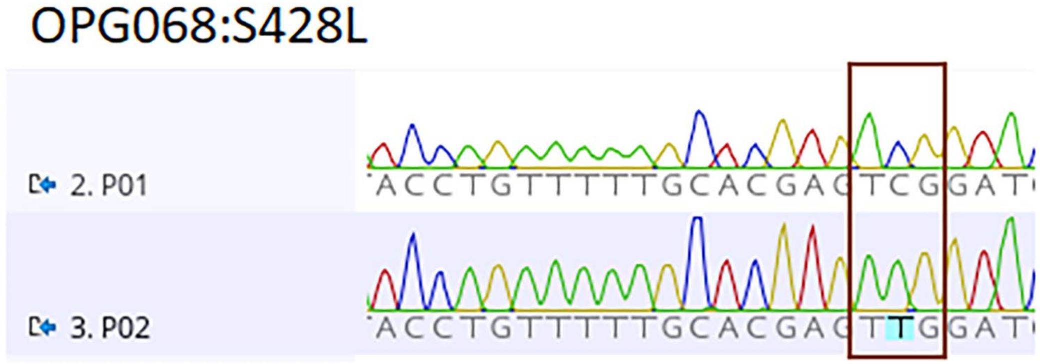

Appendix Figure 2. Representative of Sanger sequencing results confirming the presence of the novel APOBEC3-like mutation (OPG068: S428L) in the original samples.

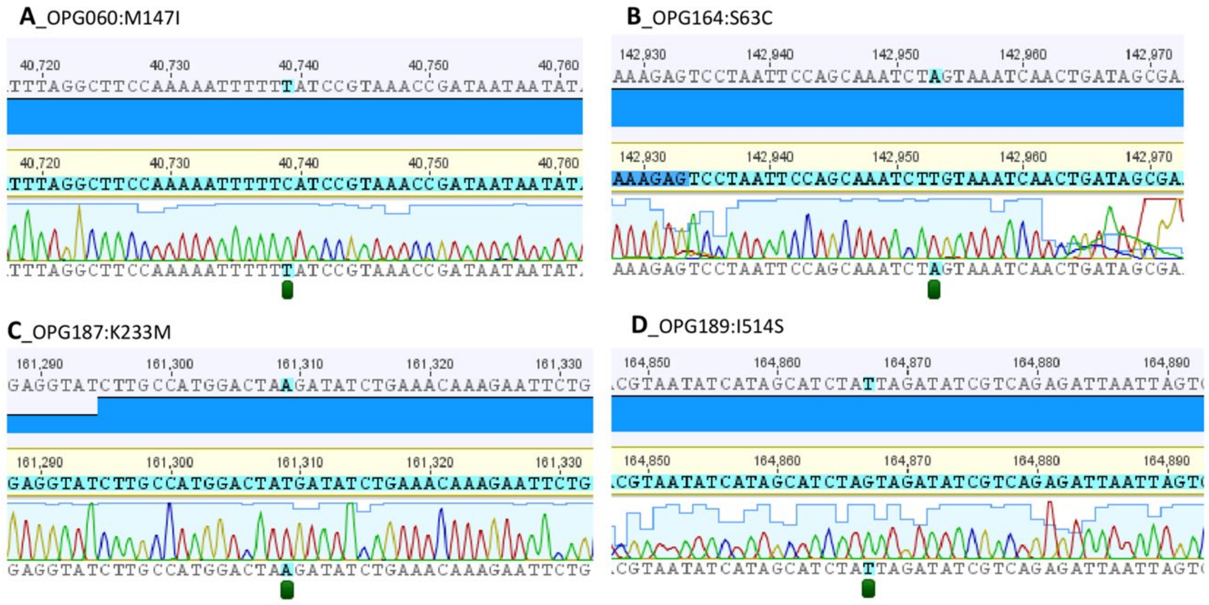

Appendix Figure 3. Screen shots showing results of Sanger sequencing reads aligned to corresponding metagenomic reads (cyan) obtained from longitudinal samples. Positions with potential nucleotide substitutions leading to amino acid changes observed in metagenomics datasets are highlighted. The discrepancy between Sanger and Metagenomics reads means substitutions were not confirmed by Sanger sequencing. Otherwise, great agreements between the two methods were achieved, especially despite low sequencing coverage obtained from metagenomics reads carrying the two nonsynonymous substitutions (Ile to Met and Ser to Cys shown in Appendix Table 7).
